# Psychostimulants and Movement Disorders

**DOI:** 10.3389/fneur.2015.00075

**Published:** 2015-04-20

**Authors:** Andres Asser, Pille Taba

**Affiliations:** ^1^Department of Neurology and Neurosurgery, University of Tartu, Tartu, Estonia

**Keywords:** psychostimulant abuse, drug induced disorders, drug abuse, movement disorders, psychostimulant toxicity

## Abstract

Psychostimulants are a diverse group of substances with their main psychomotor effects resembling those of amphetamine, methamphetamine, cocaine, or cathinone. Due to their potential as drugs of abuse, recreational use of most of these substances is illegal since 1971 Convention on Psychotropic Substances. In recent years, new psychoactive substances have emerged mainly as synthetic cathinones with new molecules frequently complementing the list. Psychostimulant related movement disorders are a known entity often seen in emergency rooms around the world. These admissions are becoming more frequent as are fatalities associated with drug abuse. Still the legal constraints of the novel synthetic molecules are bypassed. At the same time, chronic and permanent movement disorders are much less frequently encountered. These disorders frequently manifest as a combination of movement disorders. The more common symptoms include agitation, tremor, hyperkinetic and stereotypical movements, cognitive impairment, and also hyperthermia and cardiovascular dysfunction. The pathophysiological mechanisms behind the clinical manifestations have been researched for decades. The common denominator is the monoaminergic signaling. Dopamine has received the most attention but further research has demonstrated involvement of other pathways. Common mechanisms linking psychostimulant use and several movement disorders exist.

## Introduction

Psychostimulants are drugs capable of upregulating higher cortical activity and produce a transient increase in psychomotor activity. They have been used to treat a plethora of disorders including depression, obesity, nasal congestion, mood disorders, attention deficit hyperactivity disorder (ADHD), etc. However, the use of these substances has since expanded into recreational abuse in many countries.

Amphetamine, methamphetamine, cocaine, and cathinone are considered the more “classical” psychostimulants, but the group altogether comprises a much larger and constantly increasing number of substances. The main goal of synthetic substances is to mimic the psychoactive profile of amphetamine, cocaine, or other more classic drugs. Most of the abused psychostimulants are classified as illegal and thus novel synthetic drugs are becoming available to bypass the legal constraints. A large group of synthetic cathinones termed “bath salts” is becoming increasingly popular in several parts of the world (1–3).

Abuse of psychostimulants has been increasing constantly, generating more emergency room visits, hospital admissions, and lethalities each year (4, 5). In a recent Swedish study, 83% of patients admitted to an emergency room due to drug-related adverse effects screened positive for at least one psychostimulant substance (6). In UK, there was a significant increase between 2006 and 2010 in the number of individuals in an emergency department who reported the use of recreational drugs. Psychostimulants may cause serious adverse effects including neuroleptic malignant syndrome, multi-organ failure, parkinsonism–hyperpyrexia syndrome, and acute dystonic reaction. Serotonin syndrome with a high risk for a lethal outcome has been described due to intoxications after use of 3,4-methylenedioxymethamphetamine (MDMA), amphetamines, and cocaine (7, 8).

**Figure 1:**
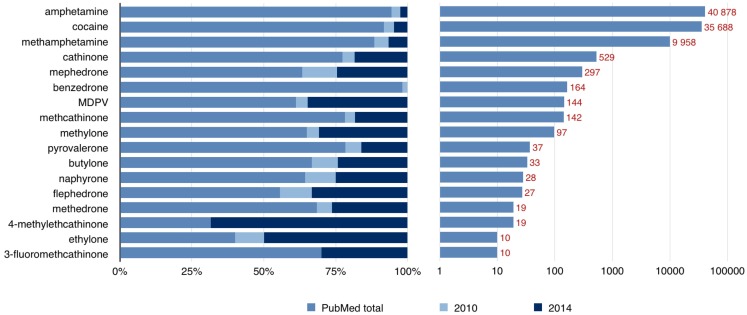
**Number of published papers available through the PubMed database by the end of 2014**. The left side of the graph shows that a large proportion of published papers concerning “new” psychoactive substances have become available only during the last 5 years (number of papers from years 2010 and 2014 are shown). The right side of the graph shows the total number of available papers on a logarithmic scale.

The pathophysiological mechanisms responsible for the effects of psychostimulants are under increasing interest. Classical substances have been studied in both humans and animals; however, the newer drugs are still not well described. In addition to classical molecular mechanisms by which psychostimulants produce their effects, including alteration of monoaminergic systems, oxidative stress, mitochondrial dysfunction, and excitotoxicity, emerging new aspects have been raised, such as neuroinflammation, blood–brain barrier damage, and neurogenesis impairment (9). There are case reports and studies available on underlying mechanisms, but a solid link is still missing between psychostimulant abuse and movement disorders. Figure 1 Lists the number of published papers on some psychostimulants and demonstrates that the data concerning newer substances has become available only in recent years.

We hereby present a literature review in order to highlight connections between psychostimulant use and movement disorders.

## Pathophysiological Aspects of Movement Disorders: A Link to Psychostimulants

Considerable overlap exists between the mechanisms influenced by psychostimulants and those involved in the pathophysiology of various movement disorders (Table 1). Movement disorders affect the control of voluntary and involuntary movements and manifest as hypokinetic or hyperkinetic disorders including parkinsonism, tremor, dyskinesias, and myoclonus. Most of these disorders are either directly or indirectly related to the basal ganglia of the brain. Evidence of altered cortical function, white matter tract involvement and widespread neural network dysfunction is also becoming available.

**Table 1:** **Examples of psychostimulants and an overview of their toxic effects mediated by monoaminergic systems**.

Chemical	Neurological adverse effects (stimulating effects not included)	Mechanism	Targets	Brain region
Amphetamine	Tremor, choreoathetosis, dystonias, dyskinesias, ataxia, gait disturbance Hallucinations Ischemic infarction, intracerebral hemorrhage	DA release and reuptake inhibition, VMAT2 redistribution; cellular toxicity due to ROS and RNS production, BBB disruption	DA, DAT, VMAT2, D2 > D1, D3 receptors, 5HT, SERT	STR, VTA, HC
Methamphetamine	Choreoathetosis, dystonias, tremor, ataxia, bruxism, seizures Behavioral disorders, punding, psychosis, depression, cognitive disorder Stroke	DA release and reuptake inhibition	DA, DAT, VMAT2, D1, D2, D3 receptors, 5HT, SERT	STR, VTA, HC
MDMA (Ecstasy)	Tremor, dystonias, parkinsonism, restless legs, bruxism Seizures Cognitive dysfunction	5HT and DA release; loss of 5HT-ergic neurons	5HT, SERT, DA, DAT	STR
Methylphenidate	Anxiety, hyperactivity, euphoria, stereotypical movements, psychiatric disturbances	DA release and reuptake inhibition, DA-ergic neuronal loss	DA, DAT, D1 receptor, NET	STR, NAc, SN, striato-orbitofrontal cortex
Cathinone	Impaired memory, depression, psychosis, insomnia, tremor, intracerebral hemorrhage	DA release and reuptake inhibition	DA, DAT, VMAT2, D2 receptor	STR
Mephedrone	Increased intracranial pressure, cerebral edema, seizures, dilated pupils Cognitive disorder Tremor, myoclonus, choreoathetosis	5HT and DA release and reuptake inhibition, 5HT and DA-ergic neuronal toxicity	5HT, DA	NAc, STR
Methcathinone (ephedrone)	Parkinsonism, limb and face dystonias, speech disorder, postural instability, gait disorder, falls	DA release and reuptake inhibition	DA, DAT, VMAT2, NET	STR
Cocaine	Tremor, tics, opsoclonus–myoclonus, dystonias, orofacial dyskinesias, parkinsonism, chorea, akathisia, restlessness Stroke Seizures	DA release and reuptake inhibition	DA, DAT	VTA, NAc, frontal and temporal cortex

*DA, dopamine; 5HT, serotonin; NE, norepinephrine; DAT, dopamine transporter; SERT, serotonin transporter; NET, norepinephrine transporter; STR, striatum; VTA, ventral tegmental area; HC, hippocampus; NAc, nucleus accumbens; SN, substantia nigra; VMAT2, vesicular monoamine transporter type 2; ROS, reactive oxygen species; RNS, reactive nitrogen species; BBB, blood–brain barrier*.

Basal ganglia are a diverse group of interconnected nuclei that serve an important part in movement execution and the relay of the associated signals. The classical model proposes a direct (striatonigral) and indirect (striatopallidal) pathway within basal ganglia involving subpopulations of striatal projection neurons (10). The circuits are activated by cortical signals and by regulating gamma aminobutyric acid (GABA) release, they eventually exert an influence on dopamine-dependent signaling and thus increase or reduce locomotor activity. Selective contributions of these pathways have been verified in animals with dopamine- and cAMP-regulated phosphoprotein Mr 32 kDa (DARP-32) loss in nigrostriatal neurons in reaction to cocaine (11).

Several neurotransmitter systems are involved with movement disorders. Altering any of these may lead to motor or cognitive disorders. The dopamine receptor system is widely spread out through the central nervous system (CNS). Basal ganglia cells harbor mainly the D1 and D2 receptors, with other receptor subtypes represented at lower levels (12). Projections of the dopamine receptor containing medium spiny neurons also show expression of specific receptor subtypes. The striatonigral pathway shows selective D1 while the striatopallidal pathway shows D2 receptor expression (13). Locomotor control is mediated mainly by D1, D2, and D3 receptors (14). Serotonin is involved in extrapyramidal motor regulation acting through serotonin receptors present in several cortical areas as well as the striatum. A number of subtypes of these receptors are known with distinctive distribution patterns throughout the brain (15). For example, several receptor subtypes (5HT1F and 5HT3A) are not found in the caudate, substantia nigra, or globus pallidus and others show either moderate or low levels of expression within the same regions in marmoset brains (16). In comparison to dopamine, serotonin system plays a less important role in motor functioning and is more involved with the cognitive functions. To some extent, serotonin can regulate dopaminergic motor function in the nigrostriatal system (17). GABA is the main inhibiting transmitter within the CNS. It exerts its effect through the GABA_A_ receptor. Sixteen subunits of the receptor have been identified throughout the CNS with a strong association with motor control (18).

Abuse of psychostimulants may cause a myriad of movement disorders through their interaction with different neurotransmitter systems, including dopaminergic, noradrenergic, serotonergic, and GABAergic systems (19). Other major molecular mechanisms include mitochondrial dysfunction, oxidative stress, and excitotoxicity, and recently suggested new phenomena of neuroinflammation, neurogenesis, and damaged blood–brain barrier (9). Chronic drug use leads to persistent adaptive changes within the reward circuitry that are associated with an impaired cognitive state and neuropsychiatric symptoms, and contribute to progression and maintenance of addiction (20).

## Psychostimulants – The “Classics” and Novel Substances, Epidemiology, and Regulations

Psychostimulants are a very diverse group of substances. The more common ones have been in routine daily use for centuries. For example, caffeine and nicotine are still parts of our daily lives. Chewing of khat leaves (cathinone), dried peyote crowns (mescaline), or using paste made of coca leaves (cocaine) was common practice to create an elevated mood, improve task performance and motor abilities among tribal members. Cocaine, amphetamine, methamphetamine, MDMA, and methylphenidate can be classified as “classic” psychostimulants. In more recent years, a number of new synthetic drugs from different substance groups have become available. In several reports, these are collectively described as novel psychoactive substances – a classification based on the stimulant effects rather than the molecular or cellular basis of action (2). Polysubstance abuse is common, with a higher risk for potential harms (21). Psychostimulants used by drivers have a serious impact on traffic safety, being associated with fatal road traffic accidents (22, 23). Stimulants used for treatment for ADHD may cause toxicity after overdose, producing major morbidity (24).

Clinically, psychostimulants cause euphoria and agitation, restlessness and hyperactivity, often stereotypical movements, anxiety, and sometimes appetite suppression. Euphoria may be followed by depression and discomfort. Sympathomimetic effects present with hyperthermia, tachycardia and hypertension, sweating, and palpitations. Neurological, cardiovascular, renal, hepatic, and psychiatric signs and symptoms are often reported as adverse effects (2, 25). Psychostimulant overdose may cause rhabdomyolysis, hypertensive crisis, malignant hyperthermia, psychosis, hyperkinetic abnormal movements, and seizures. Movement disorders may develop during acute use, chronic drug abuse, or withdrawal, and may present as transient or permanent (19, 26).

Data concerning the use of psychostimulants are not consistent. The European Union (EU) countries report on the use of amphetamines, cocaine, and ecstasy, although the reported data varies largely. High risk of designer drugs to public health has been revealed in the reports of the European Monitoring Centre for Drugs and Drug Addiction (EMCDDA), and epidemiological surveys in several populations in Europe and the United States (US) (27–29). In UK, a total of 1613 drug-related deaths were reported at 2012, and there has been a slight increase in mortality rates associated with cocaine and ecstasy, as well as novel psychoactive substances including methcathinone (30).

Studies in UK and USA have shown that despite amendments to legislation, prohibited substances are available for purchase in large quantities over the internet. New recreational substances known as “bath salts” may contain cathinones alone or in different combinations, with a high total stimulant content in some products with variable qualitative composition (31, 32).

Use of “bath salts” (synthetic cathinones) has shown an increase in recent years: there were 7467 reported cases of intoxication reported with a male to female ratio of 2.4:1 with most users being between 20 and 29 years of age. Intentional abuse is the most common reason for toxicity. A large Central London emergency medical department reported over 200 admissions due to cocaine toxicity and altogether nearly 50 cases of amphetamine and methamphetamine toxicity among all drug-related cases admitted in 2010 (33).

An overview of drug use among young people is available from the web-based Global Drug Survey. In 2013, it received nearly 80,000 responses: 11.7% of participants reported use of amphetamines during the past 12 months, 16.4% reported cocaine use, and 23.4% reported use of MDMA (“ecstasy”). Special attention was given to the use of “research chemicals and legal highs.” In countries with more than 1500 responders, their prevalence of use remained mostly between 5 and 10% (34). Despite the large number of responders, the study may not be representative of all psychostimulant due to several social issues and language barrier.

The use of psychostimulants is regulated by the 1971 United Nations Convention on Psychotropic Substances (35). It prohibits the use of cathinone and MDMA in Schedule 1, and amphetamine and methamphetamine in Schedule 2.

## Amphetamine and Methamphetamine

Amphetamine and methamphetamine are considered the prototype drugs for describing psychostimulants. Several amphetamine-like compounds exist naturally, but the first synthesis of amphetamine was likely carried out in Berlin by Lazar Edeleano in 1887 (36). It was not widely used until 1920s when the American chemist Gordon Alles resynthesized the drug and it became a treatment option for asthma. His was also the first description of the drug’s stimulant effects (37). Later amphetamine-based nasal congestion remedies became available. One of the more widely marketed was “Benzedrine,” but others were also available and used for treatment of mild depression, narcolepsy, and other disorders (38). Currently, amphetamine ranks alongside methylphenidate as the most effective drug for the management of ADHD and narcolepsy (39). Methamphetamine was first synthesized in Japan by Nagayoshi Nagai and taken into military use by several countries for its stimulating properties (40).

For the illicit drug scene, both amphetamine and methamphetamine are relatively easy to produce. Common precursors for their synthesis are ephedrine and pseudoephedrine. Illicit use has seen its ups and downs for the past 70–80 years worldwide. Amphetamines are classified as Schedule II drugs in the 1971 United Nations Convention of Psychotropic substances (35).

Amphetamine-like drugs are used as pills or capsules, powder, or fluid, and can be ingested orally, smoked, insufflated, or injected intravenously. They cause euphoria but tolerance develops rapidly. Clinically evident effects of the two drugs are nearly indistinguishable, but methamphetamine appears to be a more potent stimulant. Amphetamine and methamphetamine induce euphoria, increased energy, alertness and libido, agitation and anxiety, increased locomotor activity and stereotypical movements, as well as hyperthermia, increased heart rate and blood pressure, vasoconstriction, bronchodilatation, hyperglycemia, and suppress appetite. Psychosis, hyperkinesia, seizures, and coma have been described in emergency patients. Chronic users may develop behavioral disorders, impulsivity, punding (non-goal directed repetitive activities), hallucinations, tremor, choreoathetosis, dystonias, ataxia, and gait disturbances (41–43). Stereotyped involuntary choreoathetotic hyperkinesias are characteristic in arms, neck and head, and usually disappear during sleep, while teeth grinding (bruxism) may occur during day and night. Movement disorders may develop during abuse or abstinence, and though they a usually resolve within few days, they may remain for a long time in some cases, even after the abuse of amphetamines is stopped. Treatment with benzodiazepines or neuroleptics may be of benefit (43–45). Choreiform movements have developed as an adverse effect in the therapeutic setting of amphetamine used in the treatment of ADHD in adult and pediatric patients (46, 47).

Amphetamine and methamphetamine may cause strokes, both ischemic and hemorrhagic, probably associated with an elevated blood pressure as a major mechanism, or vasoconstriction attributed to ischemic infarction (48–50). Anxiety is one of the most prominent psychiatric symptoms in methamphetamine abusers, associated with poorer outcomes and higher levels of psychiatric symptomatology (51). Methamphetamine exposure is linked to increased rates of depression and suicide attempts (52). Stimulant drugs are known to enhance memory when processing new information but a recent study in healthy volunteers showed that dextroamphetamine in therapeutic doses increased errors during episodic memory retrieval (53). In dependent methamphetamine abusers, impairment of learning, executive functions, and information processing have been demonstrated (54, 55).

Amphetamine intoxication is an increasing burden for emergency departments: in a prospective study, amphetamine-related presentations comprised 1.2% of attendances, having a major impact to emergency rooms due to extensive resources required for patients who are agitated and aggressive, and frequently re-attend (42). A study on sudden and unexpected deaths associated with abuse of amphetamine-class drugs, demonstrated intracerebral hemorrhage, serotonin syndrome, and heart disease among causes of death based on forensic autopsies (8).

Regarding the effect to the dopaminergic system, a human [^11^C]WIN-35,428-PET study demonstrated a significant reduction in DAT density in the caudate nucleus and putamen in abusers of both methamphetamine and methcathinone (142), and an association has been found between DAT loss and methamphetamine-related psychiatric symptoms (56). Amphetamine treatment similar to that used for ADHD has been demonstrated to produce brain dopaminergic neurotoxicity in primates, causing the damage of dopaminergic nerve endings in the striatum that may also occur in other disorders with long-term amphetamine treatment (57). Through findings on the toxicity of methamphetamine toward the dopaminergic system, its link with neurodegeneration has been proposed (58, 59).

In experiments, effects of amphetamines in rodents include hyperthermia and increased locomotor activity. Amphetamine and methamphetamine act upon the CNS by altering monoamine dependent signaling. Both molecules are structurally similar to dopamine and norepinephrine. They induce dopamine release into the synaptic cleft by affecting synaptic vesicular release, more specifically vesicular monoamine transporter type 2 (VMAT2) (60), and altering DAT function by acting as substrates for the transporter (61). Concurrently, amphetamine and methamphetamine lead to serotonin and norepinephrine release by influencing the respective transporters SERT and NET (62, 63). The ability to reduce striatal DAT and SERT, functional integrity of dopamine receptors type 1 (D1) and 2 (D2) is critical (64). Synaptic reuptake of dopamine and serotonin is inhibited at higher concentrations than norepinephrine (65). Furthermore, amphetamine and methamphetamine exposure leads to production of reactive oxygen and nitrogen species further amplifying their toxic properties (66). At the cellular level, dopaminergic neuron loss within the nigrostriatal pathway has been demonstrated (67).

## 3,4-Methylenedioxymethamphetamine (Ecstasy)

3,4-Methylenedioxymethamphetamine, commonly known as “ecstasy,” was first synthesized in 1914, having been developed for a use as an appetite suppressant. It has not been used for this purpose but as a “party drug” for recreation since 1980s, being still one of the most widely used illicit drug among young adults. In a recent survey, MDMA users reported more dependence symptoms compared to users of cocaine, mephedrone, or ketamine (68).

3,4-Methylenedioxymethamphetamine is ingested orally or snorted, causing euphoria, hallucinations, anxiety, restlessness, and also gait disorders, restless legs, jaw clenching, and lack of appetite. The effects usually disappear during 24 h but in long-term use, symptoms like memory impairment, psychosis, depression, impulsivity, and anxiety may persist. Depression, memory, and concentration problems, mood fluctuation, anxiety, tremor, and weight loss have been shown to be associated with the extent of MDMA use (69, 70). Prenatal exposure to MDMA is a risk to the developing child: a prospective study demonstrated that use of MDMA during pregnancy predicts poorer infant mental and motor development at 12 months in a dose-dependent manner (71).

A range of movement disorders has been described in MDMA abusers. Abstinent addicts exhibit a large tremor during movement that may persist for months after cessation of use (72). In chronic MDMA abusers, dystonic reactions, tremor, and a syndrome with dyskinesias and stiffness have been described (73–75). Three cases of parkinsonism have been reported after chronic MDMA use, with a positive response to the dopaminergic treatment in one patient (76–78). A possible link between MDMA and Parkinson’s disease has been proposed but it has not been justified based on scientific evidence (79).

Serious complications may develop after MDMA abuse. Intracerebral hemorrhage with spastic hemiparesis (80), and two cases with aplastic anemia that resolved spontaneously (81) have been described after MDMA exposure. Severe hyperpyrexia, hyperkalemia, tremor, sweating, dehydratation, rhabdomyolysis, disseminated intravascular coagulation, and multi-organ failure may develop similarly to serotonin syndrome and neuroleptic malignant syndrome (7, 82, 83). Hyponatremia and cerebral edema have been reported as complications of MDMA use causing seizures and coma possibly leading to a lethal outcome (23, 84). A review on deaths related to MDMA in England and Wales showed that most cases with lethal outcome were reported in employed young men who typically took different drugs together with ecstasy mostly while partying (85).

3,4-Methylenedioxymethamphetamine exerts its toxic properties mainly through the serotonin system. Its presence in the CNS leads to serotonin and, to a lesser extent, dopamine release. As serotonin is a modulator for different pshychobiological functions, the toxic effects of MDMA manifest with deficits in those functions, including cognition, mood, and psychomotor skills. Neurotoxicity following MDMA is well established in animal studies showing decrease of SERT and DAT densities, with concomitant increase in extracellular serotonin and dopamine concentration (70, 86). 123Iβ-CIT SPECT imaging of human drug users has demonstrated reduction of SERT binding in the occipital cortex with the same subjects demonstrating decreased blood flow in the thalamic region on pharmacological MRI imaging (87).

## Methylphenidate

Methylphenidate is a short-acting amphetamine-like psychostimulant that was introduced for medical use in 1957. It has been used extensively in the treatment of ADHD and adult narcolepsy, and also prescribed for off-label use against depression and weight control. The most frequent adverse events have been neuropsychiatric, followed by cardiovascular and cutaneous effects (88). Methylphenidate is a Schedule II drug, considered to be medically useful but having a potential risk for abuse and dependence.

Abuse of methylphenidate has been most commonly described among students with the aim to boost energy and mental performance, improve attention and motivation, and for partying. Methylphenidate is mostly swallowed as pills or snorted intranasally (89, 90). Studies on effects of methylphenidate on cognitive function in healthy adults have controversial results, showing enhancement of cognitive performance in some experiments (91), and no significant effects in others (92). However, a functional MRI (fMRI) study in ADHD patients in a randomized controlled trial with methylphenidate showed activation of the frontal cortex and insula that are key areas of cognitive control (93).

For methylphenidate intoxication, sympathetic nervous system stimulation signs are characteristic, including hypertension, tachycardia, agitation, anxiety, psychosis, headache, and dizziness. Tremor, tics, chorea, and orofacial dyskinesias have been described as neurologic side effects of methylphenidate abuse (94, 95). When injected, methylphenidate may cause serious toxicity resulting in tissue necrosis, and occasional intra-arterial injections have lead to the amputation of fingers (96).

In children with ADHD treated with methylphenidate, tics, and orofacial dyskinesias have been described as adverse events (97, 98). Overdose of methylphenidate has caused mydriasis, tremor, movement disorders, and seizures (24), but myocardial infarction and stroke have also been reported as adverse effects at usual doses of methylphenidate for ADHD (99, 100). Rarely, lethal overdoses of methylphenidate have been reported (24, 101).

Methylphenidate belongs to the piperidine class, and its structure and effects are similar to amphetamine. In animal studies, it has increased the level of dopamine and norepinephrine through reuptake inhibition of the monoamine transporters but increase of serotonin is not critical (102). Long-term use of methylphenidate induced dopamine neuron loss that suggests neurodegenerative consequences (103). A [^18^F]FDOPA PET study in healthy subjects demonstrated that a single methylphenidate challenge increased striatal dopamine synthesis capacity, and attenuation of dopamine turnover by methylphenidate is linked to enhanced cognitive performance (104).

## Cathinones

A diverse group of substances are composed of naturally occurring cathinone and its many structural derivatives. Cathinone is naturally present in the leaves of *Catha edulis* (Khat) plants. For several centuries, humans have used natural amphetamines or cathinones – like Khat, or ephedrine from various plants of the *Ephedra* genus – for their stimulating properties. Chewing of Khat leaves has been a social and cultural tradition, and is still practiced in many African and Arabic countries, and also in Somali and Yemen communities in Europe and North America (105, 106).

“Designer psychostimulants” are often sold as “bath salts,” “plant food,” “fish food,” or “research chemicals” over the internet. Some of these substances are relatively easily manufactured without specific laboratory equipment and instructions are readily available. Cases of intoxication have been increasingly reported with new “designer” drugs. The production and abuse of cathinone derivatives is becoming a global epidemic that has raised concerns as the use of untested novel chemical substances presents potential hazards (25, 107).

The “classic” cathinones – cathinone, methcathinone, and also mephedrone – are Schedule II drugs. This has lead to the development of synthetic cathinones, also known as “legal” alternatives to illicit drugs. “Legal highs” are structurally related to amphetamine, sharing its stimulating and sympathomimetic features: excitement, euphoria, agitation, increased locomotor activity and stereotypical movements, anxiety, insomnia, hallucinations, and also creativity, productivity, and sexual arousal. Adverse effects include dysphoria, aggressiveness, psychosis, lack of concentration, lethargy and drowsiness, depression and suicidal thoughts, dizziness, and also tremor, myoclonus, and seizures (105, 108). A survey on the pediatric population with synthetic cathinone exposure showed seizure complications in 5.5% of cases (109). A rare case of intracerebral hemorrhage in a cathinone abuser has been described (110). The use of cathinones may result in cardiovascular consequences and liver or renal failure, and may lead to death in the most serious cases (111–114).

Mephedrone (4-methylmethcathinone) is one of the most popular cathinone derivatives available as a recreational drug during recent years and having a high abuse and health risk liability that has urged to classify this as a controlled substance. The most common routes of mephedrone administration are snorting and oral ingestion and also intravenous or rectal administrations have been reported. It has stimulant effects similar to MDMA and cocaine, including increased motor activity and impulsivity compulsion, psychosis, and sexual disinhibition. Several neurological side effects have been reported such as tremor, stiffness, dizziness, vision disorders, nystagmus, and sensory disorders (115–117). Cases of mephedrone toxicity with increased intracranial pressure, cerebral edema, seizures, and myoclonus have been reported (115, 118, 119). Myoclonus and seizures have also been described in methylone abusers, and fatal cases due to mephedrone or methylone toxicity have been reported, in some cases in association with multiple drug abuse, manifesting with sympathomimetic symptoms (120–122). Increasing use of the novel designer drug 3,4-methylenedioxypyrovalerone (MDPV) has been reported, resulting in neurotoxicity with manifestations of tremor, choreoathetosis, seizures, cerebral edema, dizziness, tinnitus, and headache (123–125).

The first attempts to isolate the active substance resulted in the detection of cathine (126). In 1975, cathine’s precursor cathinone was isolated and found to be the main active substance responsible for the psychoactive properties of *C. edulis* leaves. Natural Khat contains different compounds, including alkaloids, with major effects on the nervous and gastrointestinal systems. In the CNS, the target for Khat and cathinone is the dopaminergic system with widespread involvement of the nucleus accumbens while studies involving the peripheral nervous system also show effects on the serotonergic system (105, 127).

Due to the variety of different molecules their effects vary, respectively. Cathinones can be grouped by their most prominent effects on monoamine systems into (a) monoamine transporter substrates; (b) DAT-selective transporter substrates; and (c) non-substrate transporter inhibitors (25). In the pharmacology of the different cathinones, considerable differences have been found: mephedrone, methylone, ethylone, butylone, and naphyrone act as non-selective monoamine uptake inhibitors similarly to cocaine, but at the same time also induced the release of serotonin similarly to MDMA (128). However, they all have a specific pharmacological profile; mephedrone has higher brain penetration, rapid metabolism, and brain clearance than MDMA, related to high-abuse liability, but the newest compounds have not undergone thorough preclinical evaluation (117, 129). Cathinone and methcathinone act as selective catecholamine uptake inhibitors and releasers similarly to amphetamine and methamphetamine. Pyrovalerone and MDPV are potent and selective catecholamine transporter inhibitors but not substrate releasers (128). A study on monoamine-preloaded cells with a new series of designer cathinones including methedrone, pentylone, ethcathinone, pentedrone, and buphedrone, demonstrated that all the substances were potent norepinephrine uptake inhibitors but differed in dopamine vs. serotonin transporter inhibition (130). All synthetic cathinones are capable of increasing locomotor activity in animals. Other typical signs of action include hyperthermia, stereotypical behavior, and agitation, although with a different potential (129, 131, 132). Long-term cognitive and neurochemical effects of methylone and mephedrone have been shown in animal experiments (133).

## Methcathinone (Ephedrone)

Methcathinone (ephedrone) is a psychostimulant drug that is a structural analog of cathinone and methamphetamine. It can be synthesized from pseudoephedrine containing tablets available over the counter, in the presence of potassium permanganate and vinegar. Chemicals and instructions for making the drug are easily available in several languages on the internet (134). Abuse of this “designer drug” with street names Cat, Mulka, and Jeff, has become an increasing public health problem in several Eastern European countries but single cases have been described in Western and Southern Europe, and Canada (135–137). The main reason for its abuse is the amphetamine-like stimulation produced by methcathinone.

Chronic abuse of this intravenously injectable drug leads to a levodopa unresponsive parkinsonian syndrome that may develop after a few months or years of the exposure. The syndrome presents with parkinsonism, limb and face dystonias, severe speech disorders and postural instability with falls, resembling chronic manganism described in toxic conditions in welders, alloy workers, patients with chronic liver disease or hereditary conditions causing manganese overexposure (134). Antiparkinsonian treatment in these cases is ineffective and the condition may worsen progressively despite discontinuation of drug injections (137, 138).

**Figure 2:**
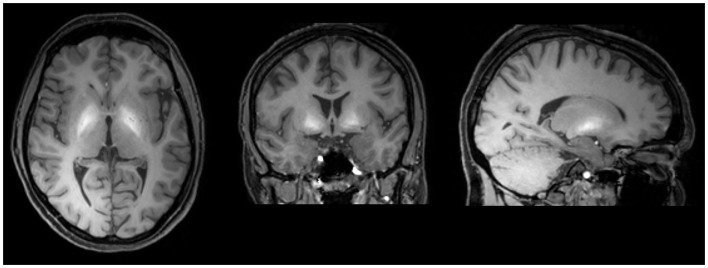
**T1 weighted MRI brain scan of a methcathinone abuser, showing high intensity areas of manganese deposits in the basal ganglia**. The deposits disappear after cessation of substance abuse while the extrapyramidal symptoms are irreversible.

In active “home made” methcathinone users, serum and hair manganese levels are extremely high. On T1 weighted MRI images of active users, symmetrical hyperintensities in the globus pallidus and substantia nigra have been demonstrated (Figure 2). Other basal ganglia including the subthalamic nucleus, putamen, caudate, and dental nucleus are less frequently involved. The increased T1 signal disappears after cessation of drug abuse though clinical manifestations are irreversible (134). On diffusion tensor imaging, widespread white matter damage has been shown in central areas and the premotor cortex (139).

This “designer” psychostimulant derived from pseudoephedrine using potassium permanganate as an oxidant, contains an excessive amount of manganese as a byproduct of the chemical reaction and has been attributed as a cause for development of the neurological syndrome (134). The role of methcathinone in the development of movement disorder syndrome is not clear but it may possibly have pathogenic effects on nigral neurons compounding the risk. Both methcathinone and manganese are capable of interacting with VMAT2. Methcathinone acts as a preferential catecholamine uptake inhibitor and a dopamine releaser similarly to amphetamine and methamphetamine. The clinical activity profile is also similar, with manifestations of euphoria, hallucinations, and motor activation (140, 141). In a human PET study, similar findings have been demonstrated in abstinent methcathinone and methamphetamine users, demonstrating a significant decrease in DAT density in caudate nucleus and putamen (142). In animal models, multiple administrations of methcathinone have caused a persistent deficit in dopaminergic system (128, 143). However, a divergence between *in vitro* and *in vivo* properties of methcathinone has been demonstrated: the drug is very selective and potent releaser of the catecholamine transporters *in vitro*, but elevates both dopamine and serotonin levels *in vivo* (107).

## Cocaine

Cocaine is a potent “classic” psychostimulant. The natural source of cocaine in the form of coca plant leaves has been known for centuries. In 1859, an active alkaloid cocaine was isolated by Albert Niemann of Germany (144). For the following half a century, it was mostly used for medical purposes as a local anesthetic and treatment of depression but thereafter it became increasingly popular as a drug of abuse. Cocaine is a Schedule II drug.

Clinically evident effects of cocaine are largely dose dependent with correlations to plasma levels. At the same time, large differences between individuals are present. Clinical manifestations may vary by the route of administration, purity of sample, and duration of cocaine abuse. Its stimulant effects manifest as agitation, euphoria, and hyperthermia (145). A variety of movement disorders have been reported in association with cocaine abuse. Slow frequency (<8 Hz) hand tremor has been described in abstinent cocaine abusers (146). Cocaine may induce tics or exacerbate Tourette’s syndrome (147, 148), and it has also been reported to cause punding and opsoclonus–myoclonus (149). Dystonic reaction has been reported in children after accidental exposure to cocaine in their home environments (150). Neuroleptic malignant syndrome following delirium has rarely been described in the acute stage, as well as persistent parkinsonism following a 3-month abstinence from the drug (151). A rare syndrome of fulminant encephalopathy with manifestations of seizures, bradykinesia, myoclonia, and bilateral MRI hyperintensities in basal ganglia has been described in HIV-positive cocaine abusers (152). The use of potassium permanganate in the processing of coca-leaf extraction can also lead to manganese intoxication (153).

Neurological complications are more common and severe with the smokeable alkaloidal form of cocaine known as “crack.” Cocaine exposure has been reported to cause reversible choreiform limb movements with restlessness or akathisia, and orofacial dyskinesias, referred to as “crack dancing” (154, 155). Usually, choreiform or dystonic movements last from minutes to few days (156, 157), but a case with long-term abnormal movements persisting after a 20-month abstinent period has been described (158).

Cocaine is the most frequent drug of abuse associated with cerebrovascular events occurring as a result of cerebral vasospasm, vasculitis, cardiac arrhythmia, increased platelet aggregation, or hypertension associated with cerebral autoregulation. Cocaine may cause ischemic or hemorrhagic strokes by both nasal insufflation and alkaloidal “crack” smoking routes, but there are a higher proportion of hemorrhages with nasal use (145). Cardiotoxicity has been a frequent cause for sudden or unexpected death associated with cocaine as demonstrated by autopsy findings like coronary artery disease, enlarged heart, and myocarditis (159).

A recent study has provided evidence of the disruptive effects of cocaine on stimulus–response learning and episodic memory (160). Cognitive impairment has been associated with connectivity changes in fMRI (161). Prenatal cocaine exposure due to substance use among pregnant women has been shown to affect development, behavioral outcomes, and motor performance in children (162, 163).

Radiological studies of abstinent cocaine abusers demonstrate increased availability of DAT (164). This may hint at a compensatory upregulation of the dopaminergic system in response to hindered DAT function in the setting of chronic cocaine abuse. D2 and D3 receptor availability is decreased in early abstinence and is also present at 4 months after cessation of drug use (165) indicating at a possible permanent structural damage of the dopamine system. Other means for assessment of structural changes induced by cocaine use include different MRI-based techniques. Diffusion tensor imaging reveals that white matter pathways in the frontal lobes of the brain are altered, thus reducing connectivity between brain areas (166). MRI-based volumetric assessment of both white and gray matter in the frontal areas also has shown a decrease in chronic cocaine users (167). fMRI studies of chronic cocaine abusers have demonstrated a significant motor function deficit associated with alterations to the dorsal striatum, and an impaired cortical–striatal connectivity that suggests a fundamental deficit of cognitive processing (161). Structural abnormalities showing compromised white matter integrity in cocaine dependence have been associated with functional impairment in decision making (168).

The main effects of cocaine are produced by its influence on the dopamine and serotonin signaling, although other systems are involved. Cocaine leads to increased dopamine release, an effect shown to be age dependent (169), with extracellular serotonin concentrations also increased (170). Monoamine release is facilitated by cocaine DAT and SERT blocking abilities with similar binding affinities thus blocking monoamine reuptake (171). A neuropathological study in chronic cocaine users demonstrated a threefold increase in α-synuclein levels in the dopamine cell groups of the substantia nigra and ventral tegmentum (172).

Serotonergic system dysfunction plays an important role in cocaine sensitivity. SERT and serotonin-1A receptors have been associated with increased self administration and locomotor activity in rats. To a greater or lesser extent other serotonin receptor subtypes are also influenced (173).

## Conclusion

Psychostimulants are gaining attention as a research subject due to their popularity among recreational users as the number of adverse effects increases. The number of substances regarded as psychostimulants is continuously increasing with new molecules constantly being added to the nomenclature.

The main effect of these drugs is based on various alterations they induce in the monoamine systems. The major changes that are induced include synaptic monoamine release, inhibition of their reuptake, and changing the signaling that is dependent on respective transporters. These alterations are mostly reversible when administration of the causing agent is discontinued. Nonetheless, there are reports of more permanent damage at molecular and cellular levels that persist after drug use has ended. As more evidence becomes available, the pathological alterations behind the mechanisms will be better understood.

In addition to agitation, increased locomotor activity, euphoria, and other psychiatric disturbances, psychostimulant use may lead to a variety of acute movement disorders: tremor, gait disturbances, parkinsonism, and various hyperkinetic disorders including chorea, dyskinesias and dystonias, myoclonus, and akathisia. Acute syndromes are often witnessed by emergency medical departments and intensive care units. In acute onset movement disorders particularly in young people, illicit drug use should always be contemplated as a possible secondary cause. Failure to recognize these disorders can lead to missed therapeutic opportunities and occasional fatalities.

At the same time, psychostimulant related chronic movement disorders are relatively infrequent. Psychostimulant drugs can exert a deleterious effect in brain areas also altered in Parkinson’s disease and dystonias. The prognosis of long-term side effects of recreational drugs may be poor, causing irreversible disability. Spreading abuse of psychostimulants including new psychoactive designer drugs is a serious public health concern, and physicians must be aware to recognize these disorders within the social risk groups.

## Conflict of Interest Statement

The authors declare that the research was conducted in the absence of any commercial or financial relationships that could be construed as a potential conflict of interest.

## Abbreviations

ADHD, attention deficit hyperactivity disorder; CNS, central nervous system; DARP-32, dopamine- and cAMP-regulated phosphoprotein Mr 32 kDa; DAT, dopamine transporter,; EMCDDA, European Monitoring Centre for Drugs and Drug Addiction; EU, European Union; fMRI, functional magnetic resonance imaging; GABA, gamma aminobutyric acid; MDMA, 3,4-methylenedioxymethamphetamine; MDVP, 3,4-methylenedioxypyrovalerone; MRI, magnetic resonance imaging; MRS, magnetic resonance spectroscopy; NET, norepinephrine transporter; PET, positron emission tomography; SERT, serotonin transporter; SPECT, single photon emission computed tomography; UK, United Kingdom; USA, the United States of America; VMAT2, vesicular monoamine transporter type 2.
